# The Anti-Metastatic nm23-1 Gene Is Needed for the Final Step of Mammary Duct Maturation of the Mouse Nipple

**DOI:** 10.1371/journal.pone.0018645

**Published:** 2011-04-07

**Authors:** Camille Deplagne, Evelyne Peuchant, Isabelle Moranvillier, Pierre Dubus, Sandrine Dabernat

**Affiliations:** 1 Unité U1035, INSERM, Bordeaux, France; 2 Biothérapies des maladies génétiques et cancers, Univ. Segalen Bordeaux, Bordeaux, France; 3 Équipe 2406 Histologie et pathologie moléculaire des tumeurs, Univ. Segalen Bordeaux, Bordeaux, France; Roswell Park Cancer Institute, United States of America

## Abstract

Nm23/NDP kinases are multifunctional enzymes involved in the general homeostasis of triphosphate nucleosides. Numerous studies have shown that NDPKs also serve as regulatory factors of various cell activities, not always connected to nucleotide phosphorylation. In particular, the nme-1 gene, encoding the NM23-1/NDPKA protein, has been reported as a metastasis suppressor gene. This activity was validated in hepatocellular tumors induced in nm23-1 deficient mice. Yet, data describing the primary physiological functions of nm23-1/NDPKA is still scarce. We have characterized in depth the phenotype of nm23-1 deletion in the mammary gland in mice carrying whole body nm23-M1 invalidation. We also asked why the nm23-M1^−/−^ mutant females displayed severe nursing disability. We found that the growth retardation of mutant virgin glands was due to reduced proliferation and apoptosis of the epithelial cells within the terminal end buds. The balance of pro/anti-apoptotic factors was impaired in comparison with wild type glands. In the lactating glands, the reduced proliferation rate persisted, but the apoptotic factors were unchanged. However, those defects did not seem to affect the gland maturation since the glands lacking nm23-1/NDPKA appeared morphologically normal. Thorough examination of all the functional aspects of the mammary glands revealed that lack of nm23-1/NDPKA does not impact the production or the ejection of milk in the lumen of lobuloalveolae. Interestingly, an epithelial plug was found to obstruct the extremity of the unique lactiferous duct delivering the milk out of the nipple. These cells, normally disappearing after lactation takes place, persisted in the mutant nipples. This work provides a rare instance of nm23-1/NDPKA physiological functions in the mammary glands and reveals its implication as a modulator factor of proliferation and apoptosis in this tissue.

## Introduction

Nucleoside diphosphate kinases (NDPKs) are ubiquitous enzymes synthesizing nucleoside triphosphates. They are encoded by 9 genes in humans, forming the family of the non metastatic #23 (nm23) genes or nme genes. They can be split into two groups according to their levels of homology with the historical isoforms nm23-1/NDPKA and 2/NDPKB [1]. They have first been considered as housekeeping enzymes until the gene nm23-1/nme1 was found involved into the mammary gland metastatic process [2]. Since then, nm23/nme genes have been implied into various and critical cell functions, mainly linked to tumor transformation, progression and dissemination [3], [4].

During the early stages of tumor formation, nm23-1 and -2 genes are overexpressed, then the loss of the nm23 signal is correlated with higher tumor aggressiveness such as in mammary carcinomas or in melanomas [4]. Importantly, the anti-metastatic activity of nm23-1 has been validated in hepatic tumor models in the mouse [5]. Several explanations have been raised to explain nm23 implication as a tumor suppressor. Among them, it seems that nm23 genes products interact with cytoskeleton elements leading to modulation of cell/cell and cell/extracellular matrix bonds and they indirectly regulate small G protein activity such as Rac1 or Rho [6], [7], [8] leading to motility inhibition. It has also been demonstrated that nm23-1/nme1 regulates the cell surface expression of integrin receptors and matrix metallo-proteases, and thus directly controls the cell adhesion machinery [9].

Null mutation in the awd gene, the unique NDPK coding gene in Drosophila Melanogaster causes lethality in larvae [10]. Bacteria and yeasts lacking NDPK encoding genes live normally [11], [12], although the E. Coli model shows a high genomic mutations incidence [13].

NDPK A and B mouse proteins shares >98% identity with their human counterparts thereby rendering the mouse as a valuable model to explore the nm23 genes functions. Nm23-M1 invalidation has been carried out to study its physiological functions [14]. Lack of NDPKA does not result in major deficiency; the animals develop normally and are fertile. However, they display reduced body weights. More importantly, newborns' survival is highly compromised when the mother lacks both nm23-M1 valid alleles as shown by cross-fostering experiments. The invalidation of nm23-M2 gene has not been reported. However, the double knock out of nm23-M1 and nm23-M2 genes (encoding the mouse NDPKA and NDPKB, respectively) was facilitated by the localization of both genes on the same chromosome about 5000 base pairs apart [15]. The double mutants die perinatally with severe hematologic disorders such as anemia, defective erythroid cell terminal maturation and abnormal iron metabolism [16]. To date, no phenotype in the mammary glands was explored in this model.

Numerous studies, most of them from Pat Steeg's group [17], suggested that nm23-1 expression was linked to the control of the mammary gland development. Nm23-M1 was detected in the mouse embryo at 12.5 dpc in mammary gland buds and later during development, in the epithelial tree as well as in the terminal end buds (TEBs) [14]. In addition, mammary gland growth retardation was evidenced in the absence of nm23-M1, in virgin females aged between 5 and 15 weeks old. However, gross morphological differences were abolished during gestation and later during lactation. Whole mount analyses showed that the growth retardation observed in virgin females is not anymore visible during pregnancy and after birth [14].

Thus, besides its well-known implication in breast cancer metastasis formation, the nm23-1 gene seems to impact the mammary gland development. However, the molecular routes involved have not been explored yet. This study was aimed to provide a more detailed analysis of nm23-M1 invalidation in the mammary gland. We present data related to the effect of the lack of nm23-M1 on mammary gland cell proliferation and apoptosis in the virgin females and we propose an explanation for the high mortality rate of pups born from homozygous mutant mothers.

## Materials and Methods

### Generation and maintenance of transgenic mice

All animal studies adhered to protocols approved by the University of Bordeaux 2 animal care and use committee and the commission de genie genetique (Direction Generale de la Recherche et de l'Innovation). The generation of the nm23-M1 KO mice (nm23-M1^−/−^) was already described [14]. Mice were maintained at the University Bordeaux 2 animal facility according to the rules enforced by the Institutional Animal Care and Use Committee, in EOPS conditions. Mice used in this study are from the C57BL/6J genetic background. Noteworthy, the nm23-M1^−/−^ mice exist in the 129/SV genetic background. We did not perform experiments on this background. However, it is likely that similar observations could be done, keeping in mind that the penetrancy of the phenotype is slightly lower in the 129/SV background as compared to the C57BL/6J.

### Mammary gland processing and staining

Mouse mammary glands were retrieved and processed according to the following protocol: the thoracic glands were used for RNA extraction and the abdominal glands were used for histology, immunodetection and protein extraction. The cervical and inguinal glands were not used in our study.

For histology procedures, glands were fixed in 10% NBF, embedded in paraffin and processed by routine histology procedures. Beta-galactosidase was detected as already described [14]. For immunohistochemistry, the following primary antibodies were used: mouse anti-alpha actin (1/500, Sigma), mouse anti-BrDU (1/1000, Dako), rabbit anti-oxytocin receptor (1/200, Abcam). Appropriate anti-rabbit or anti-mouse biotinylated secondary antibodies in DAKO EnVision kits (DAKO) were used to detect the presence antibody staining. For immunofluorescence staining, anti-mouse–FITC (Vector Laboratories) was used. Apoptosis was detected by using an In Situ Cell Death Detection kit (Roche). Bromodeoxyuridine (BrdU, Sigma) labeling was initiated by intraperitoneal injection (100 ug/g body weight) the day before the sacrificing of the treated animals. For morphometric analyses 10 sections were scored on at least five fields from the WT group (n = 3) and nm23-M1^−/−^ group (n = 4). Results are expressed as mean of positive BrdU/total nuclei of epithelial cells ± SD.

### Nipples collection and processing

Nipples from WT, heterozygous and mutant females were retrieved either in 6 weeks old and 12 weeks old virgin females or 3 days after delivery and fixed in 10% NBF. Transversal or longitudinal serial sections were carried out and stained with Hematoxilin and Eosin or Masson stain that reveals connective tissue in green. They were also processed for immunohistochemistry as described above and stained with mouse anti-PCNA antibody (Cell Signaling), rabbit anti-betagalactosidase antibody (Invitrogen), mouse anti-cytokeratin 2E (Acris antibodies), mouse anti-keratin 10 and mouse anti-keratin 14 (both from San Cruz Biotechnologies).

### Western-blotting and PCRs

Mammary glands were washed with PBS, homogenized and sonicated in a RIPA buffer and processed for western blotting. Membranes were incubated with rabbit anti-phospho AKT, anti-BAX, anti-BCL-XL, anti-phospho STAT3, anti-phospho STAT5 (all from Cell Signaling Technologies) and anti-oxytocin receptor (Abcam). Rabbit anti-actin antibody (Cell Signaling Technologies) was used to assess equal loading of the samples. Primary antibodies were detected with specific anti-rabbit- or anti-mouse-IgG-HRP (Cell Signaling Technologies). Proteins were visualized using the ECL detection system (Amersham Pharmacia Biotech).

Total RNAs were extracted from mammary glands in TRIZOL solution (Invitrogen). Reverse transcription was carried out on 1 ug of total RNAs using the Superscript III-RT kit according to the manufacturer recommendations (Invitrogen). PCRs were performed with the PCR Master Mix (Promega) on 1/10^th^ of the RT. Primers were designed as follows: beta-casein sens : 5′CTTGCTAATCTGCACCTTCC3′, Beta casein antisense 5′AGAGTCCATGGGTCGAATTC -3′, Lactoferrin sens : 5′GCTGTAGCAGCAGTTAGAAG3′, Lactoferrin antisense : 5′ACTGAACCTGTTGGTCAAGC3′, OTR sens : 5′CAGGTGCACATTTTCTCGCT3′, OTR antisense : 5′GAGCATGTAGATCCATGGGT3′.

### Statistical analyses

Statistical significance was determined using unpaired Student's *t*-test. Results presented as mean+/-SD were considered significant when p≤0.05.

## Results

### Nm23-M1 is expressed in the luminal cells of the mammary gland epithelium

When we first described nm23-M1 invalidation in the mouse, we observed mammary gland growth retardation in both ductal elongation and branching, in the virgin females [14]. We also mentioned that nm23-M1 was present in the epithelium, at the macroscopic level. Nm23-M1 invalidation was carried out so that a reporter gene (LACZ) was introduced in place of the disrupted gene. Consequently, the bacterial beta-galactosidase is expressed under the control of nm23-M1 promoter. To identify the cells expressing nm23-M1, we now performed beta-galactosidase detection in mammary gland sections in nm23-M1^+/−^ mice. Beta-galactosidase was evidenced in the luminal epithelium of the mammary ducts from 6 week old virgin females, whereas the surrounding myoepithelial cells appeared negative. The adipose tissue showed no signal (figure S1a). This result was further confirmed when sections from lactating mammary glands were analyzed at day 1 after the birth, which will be further mentioned as L1 (figure S1b). Beta-galactosidase was evidenced in the epithelial compartment of the lobuloalveolar structures.

### Lack of nm23-M1 results in diminished proliferation and apoptosis

**Figure 1:**
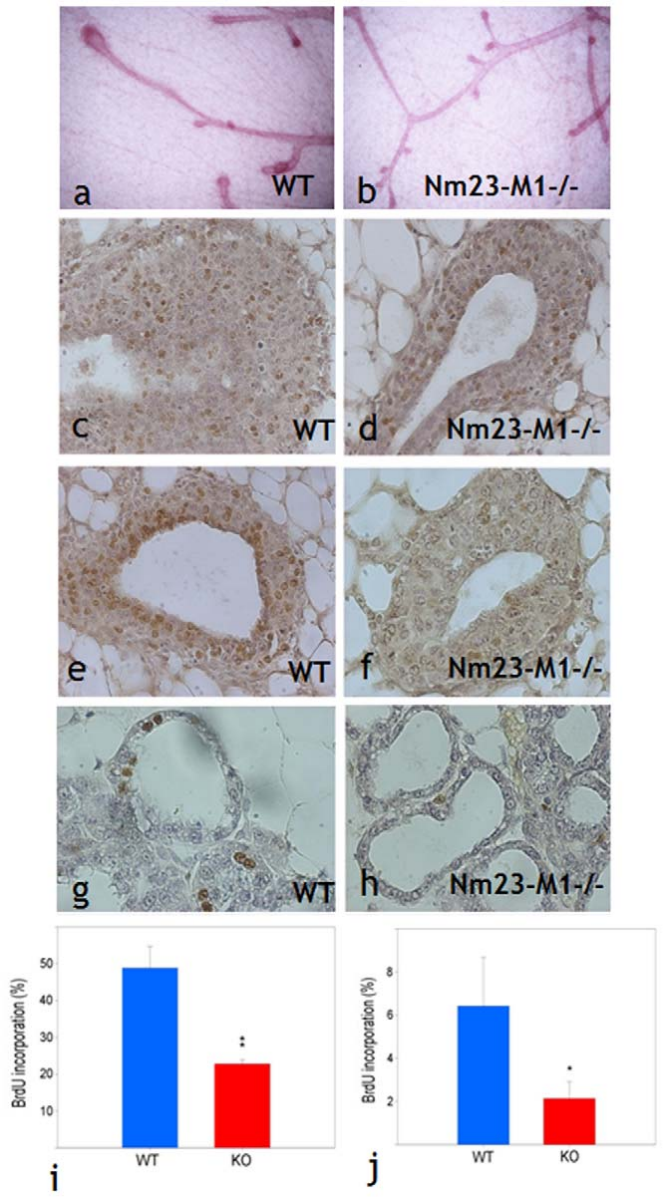
Lack of nm23-M1 results in impaired proliferation. Whole mounts of WT (a) and nm23-M1^−/−^ (b) mammary glands show a delayed growth of the TEBs in the mutant glands (original magnification X5). BrdU incorporation (brown nuclei) was evidenced in sections from mammary glands from 6 weeks old WT (c,e) or nm23-M1^−/−^ (d,f) virgin females and L1 WT (g) or L1 nm23-M1-/- (h) lactating females (original magnification X400). Quantifications in virgin glands (i) and lactating glands (j) revealed a drop in BrdU incorporation in the epithelial cells of the mutant glands. KO: nm23-M1^−/−^.

As already mentioned above, nm23-M1^−/−^ virgin females showed delayed mammary gland growth. Indeed, mammary ducts of the nm23-M1^−/−^ mice failed to extend throughout the mammary fat pad [14]. In the mouse, the mammary gland development is under the control of structures located at the end of the branches of the mammary tree. These transient structures, called Terminal End buds (TEBs), appear at 3 weeks of age and undergo important cell proliferation. They later become terminal ductal structures with low mitotic activity and intensive apoptosis leading to the formation of the lumen of the ducts [20]. To assess whether the growth retardation observed in nm23-M1^−/−^ mice was due to impaired proliferation or apoptosis, microscopic examinations were carried out. The observation of the mammary tree of 3 weeks old virgin mice revealed substantial alteration in the aspect of nm23-M1^−/−^ TEBs (Figure 1a, and 1b). WT structures seemed to undergo terminal changes including the lumen formation of the future lobuloalveolar structures. Nm23-M1^−/−^ TEBs on the contrary appeared smaller and the lumen was not detectable. Consequently, histometric analysis was performed on 6 weeks old females, where lumen was easily evidenced in each group of mammary glands (Figure 1c to 1f). At that stage, TEBs seemed to be morphologically normal in nm23-M1^−/−^ mice. However, the number of BrdU positive cells was decreased by about 2-fold in the nm23-M1^−/−^ mice as compared to the WT mice (Figure 1i n = 3 in each group and p<0.01). As already mentioned, mammary growth retardation seemed to be rescued during pregnancy and in nursing females. Sections from L1 lactating mammary gland showed less BrdU incorporation in WT glands as compared to the 6 weeks old WT virgin glands (Figure 1g and 1h). The diminished proliferation observed in the nm23-M1^−/−^ virgin glands persists in the L1 gland (Figure 1j) with the same fold difference (n = 3, p<0.05). This defect though, is not sufficient to disrupt the growth of the gland, since the morphology of the epithelium appeared normal in the nm23-M1^−/−^ glands (see below).

**Figure 2:**
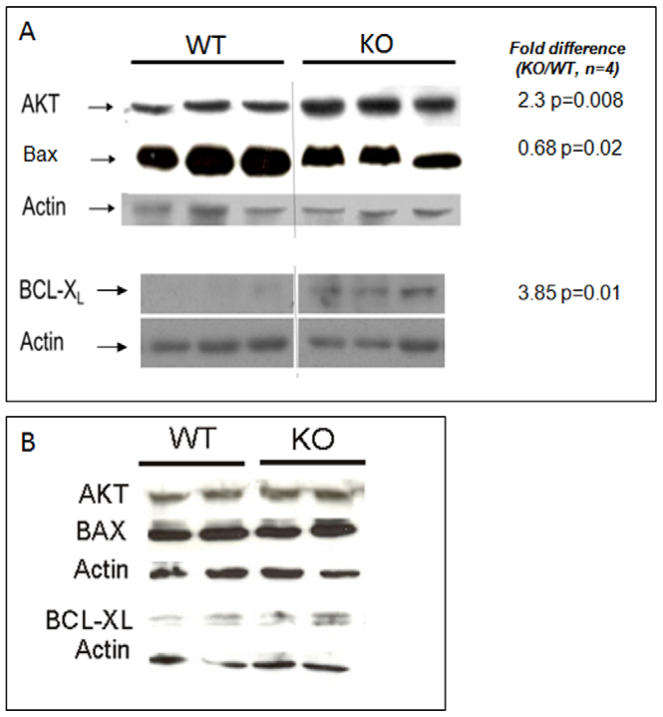
Lack of nm23-M1 results in diminished apoptosis markers in the virgin but not the lactating mutant glands. A: protein extracts from 6 weeks old virgin WT and nm23-M1^−/−^ (KO) glands were processed for western-blotting. Images were analyzed by densitometry to measure intensity differences between the two groups. Results are indicated on the right hand side. B: protein extracts from L1 lactating WT and nm23-M1^−/−^ (KO) glands were processed for western-blotting. No difference in band intensity was evidenced between the two groups.

In the virgin glands, ductal morphogenesis is controlled by apoptosis undergoing in TEBs. This process involves the activity of Bcl2 family members such as Bcl-XL and Bax [21] and PKB/AKT [20] for example. In 6 weeks old nm23-M1^−/−^ glands, apoptosis tended to be diminished when sections were observed, although it was difficult to quantify this phenomenon, whereas in L1 lactating glands, no obvious differences in the density of apoptotic bodies could be evidenced (data not shown). Therefore, we performed western-blot analyses testing for the above cited factors. We found that anti-apoptotic factors were increased in nm23-M1^−/−^ virgin glands protein extracts as compared to WT, whereas proapoptotic factors were diminished (Figure 2A). However, when L1 glands were examined for the same factors, no difference was seen (Figure 2B).

Thus, the mammary glands from nm23-M1^−/−^ virgin females display delayed growth, which can be accounted for reduced proliferation and apoptosis. However, in the lactating glands, the differences observed in the virgin glands are not enhanced (proliferation) or are absent (apoptosis).

### Lack of nm23-M1 results in severe nursing deficiency

As already mentioned above, the growth retardation observed in nm23-M1^−/−^ virgin females seemed to be rescued in the pregnant females, or right after birth since whole mount glands did not show striking morphological differences [14]. We looked at lactating glands removed at day 1 after the birth of the pups (L1) since feeding did not take place. Indeed, newborn from nm23-M1^−/−^ mothers failed to accumulate milk in their stomach (Figure S2). Conversely, this phenomenon is easily observable in pups fed by WT mothers. Over 13 litters obtained from mutant mothers for this study, 11 litters died within the first 2–3days (85%) where only 5 of 14 of the WT litters died (35%). The weight of the mutant pups was in average significantly lower as compared to the WT pups at L1 (1.34g±0.12 for mutant and 1.68g±0.16 for WT, p = 0.02), likely because they were not fed.

**Figure 3:**
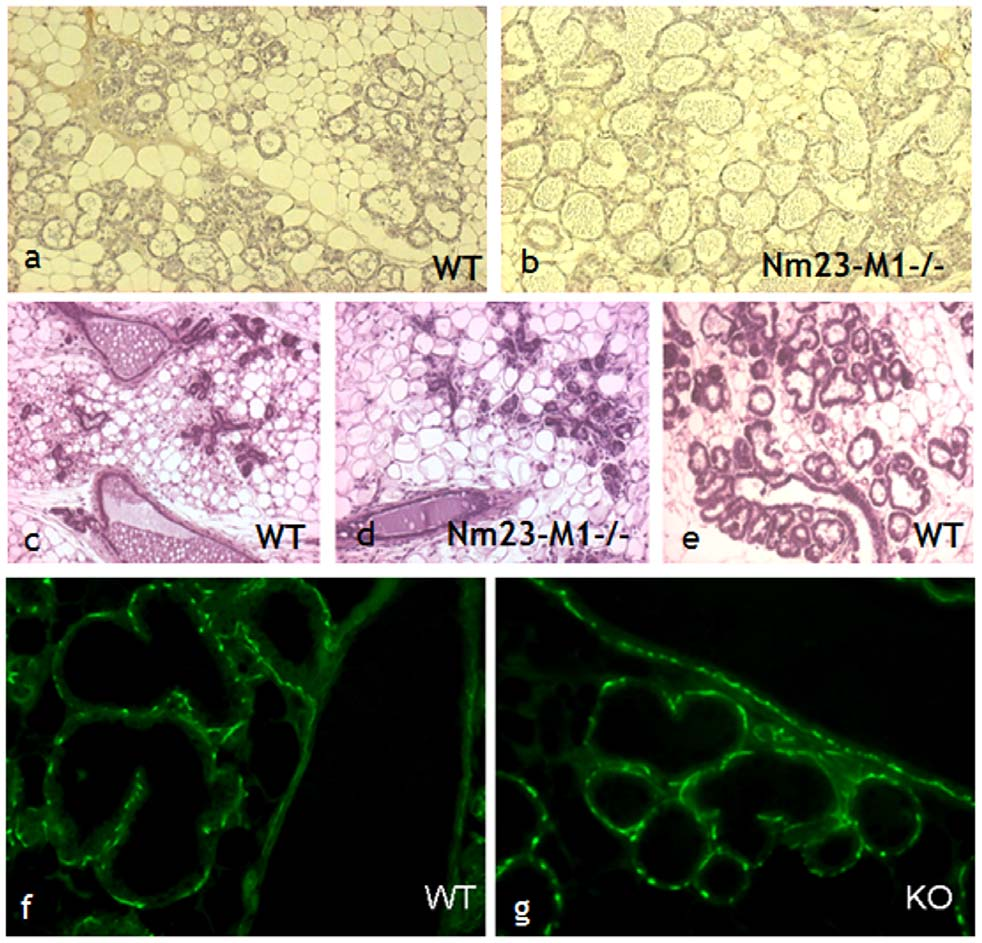
The morphology of lactating L1 and L3 glands seems normal in mutant females. Sections from lactating L1 WT (a) and nm23-M1^−/−^ (b) mammary glands do not reveal any abnormality. L3 nm23-M1^−/−^ glands (d) show signs of involution as evidenced by the diminished density of lobuloalveolar structures, not seen in L3 WT lactating gland (e). However, L3 glands from WT females from which the litters have been removed (c) do not appear different from nm23-M1^−/−^ L3 gland (Original magnification X200). Sections have been stained with anti-alpha actin to reveal normal pattern of myoepithelial cells in WT (f) and mutant (g) L1 lactating glands (Original magnification X400).

Further examination of sections from L1 glands did not reveal obvious differences in the morphology of the mammary epithelium, but we often observed distended lobuloalveolar structures with accumulation of secretion in the lumen (Figure 3a and b). As nm23-M1 seemed to be involved in apoptosis, we sought to determine whether mammary gland involution was affected in the nm23-M1^−/−^ females. The morphological examination of L3 glands showed intense reduction of the number of lobuloalveolar structures in the mutant glands as compared to feeding L3 WT glands (Figure 3d and e). This was accompanied by a significant drop of the mammary gland weight in mutant mice as compared to WT (0.119±0.04 mg and 0,248±0.05 mg, respectively, n =  8, p<0.001). However, we looked at glands from WT females which had not been feeding because their pups had been taken away and we didn't see any morphological difference with the mutant glands and the weights were similar in both groups (Figure 3c and 0.113±0.02 mg, p>0.05). Furthermore, at the molecular level, we looked at the activation of STAT3, a major actor of the mammary gland involution [20]. We also looked at the levels of caspase 3. No difference was seen in the mutant glands, compared to the non feeding WT glands at L1 or L3 (data not shown). Together with the fact that we did not see any difference in the balance of Bcl2 family members in post-birth glands (not shown), this shows that early involution of the gland cannot explain the fact that the majority of nm23-M1^−/−^ females do not feed their pups.

### The severe feeding deficiency is not related to a functional defect in the mutant gland

**Figure 4:**
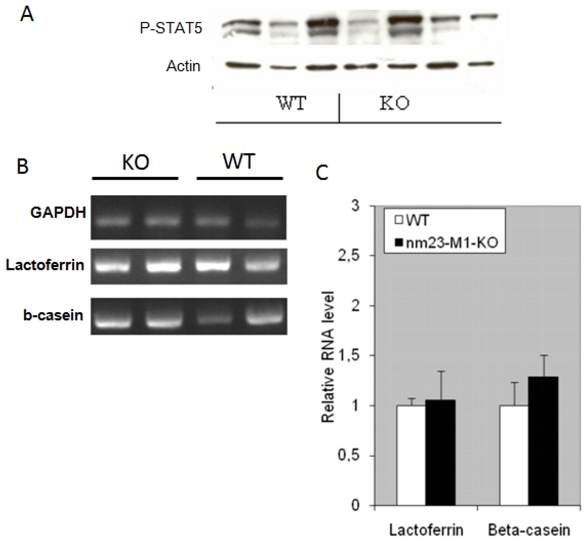
Mutant mammary glands do not present functional defects. Western-blots have been carried out to detect the level of phospho-STAT5. Actin was detected to check on sample loading. (A) RT-PCRs have been performed and quantified by densitometry. RNA levels related to GAPDH signal are reported (C).

Milk secretion is under the control of a major hormonal actor: the prolactin [22]. We looked at the activation of STAT5, which is directly under the control of the prolactin receptor activation. Phospho-STAT5 was detected in L1 gland protein extracts by western-blotting (Figure 4A). Again, densitometric analysis failed to demonstrate a deficient STAT5 activation in the mutant glands. Furthermore, we confirmed that milk was produced in the mutant glands by detecting RNAs encoding milk proteins in both WT and nm23-M1^−/−^ glands, at similar levels (n = 4 in the WT group and n = 5 in the mutant group, Figure 4B and C).

The milk ejection reflex is dependent upon the release of pituitary oxytocin and its interaction with a specific receptor (Oxytocin receptor, OTR) within the mammary gland [23]. The ejection is also mediated by the contraction of myoepithelial cells surrounding the ductal/glandular epithelium. We first attempted to inject oxytocin in post-partum females. This failed to rescue normal feeding of the pups, suggesting that lack of oxytocin was not the cause of the deficient feeding. When OTR expression was examined by immunohistochemistry, no abnormality was seen in the distribution of the receptor in the mammary gland of mutant mice (Figure S3a to d). In the same way, myoepithelial cells were easily evidenced using anti-alpha actin antibody, with normal position and density in mutant glands as compared to WT (Figure 3f and g).

Thus, nm23-M1^−/−^ mammary glands produce milk and display the hallmark of normal milk secretion and ejection.

### Lack of nm23-M1 results in abnormal maturation of the mammary glands terminal lactiferous ducts

**Figure 5:**
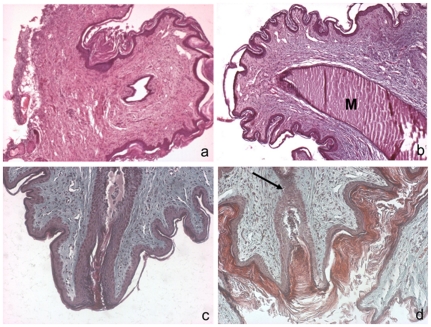
Nipples from nm23-M1^−/−^ mammary glands are obstructed. Sections from WT (a, c) and nm23-M1^−/−^ (b, d) nipples were sectioned and stained by hematoxylin and eosin (a, b) or trichrom Masson (c, d). The general architecture of dermis and epidermis of the mutant nipple doesn't seem overly changed. However, close examination revealed that the final opening of the lactiferous canal is obstructed by epithelial cells in the mutant glands (arrow). M: milk. Original magnification X200 (a, b) X400 (c,d).

**Figure 6:**
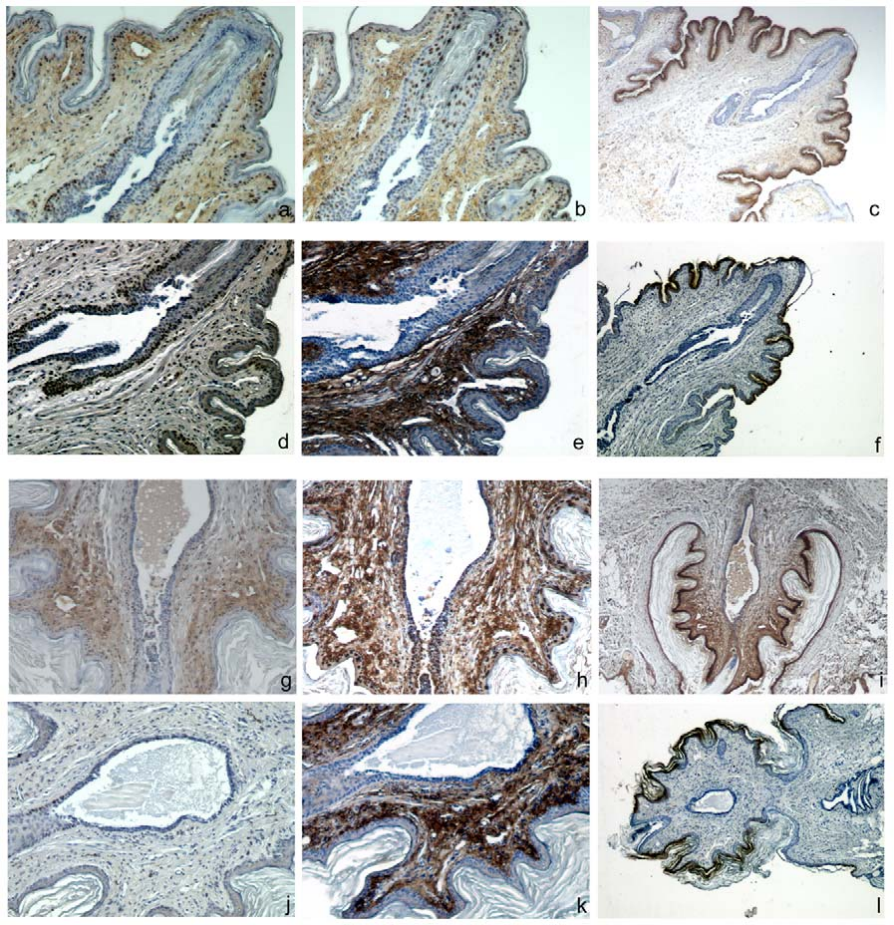
Proliferation and elimination of the epithelial plug does not occur in the lactiferous sinus of nipples from females which are not nursing. Serial sections of nipples were stained with anti-PCNA (a, d, g and j), anti-beta-galactosidase (b, e, h and k) and anti-K2e (c, f, i and l). Sections from mutant (a, b and c) and WT (d, e and f) nipples from nursing females are fully opened at the edge and show numerous PCNA-positive cells within the wall of the lactiferous sinus. Sections from mutant (g, h and i) and WT (j, k and l) nipples from non nursing females show a persisting epithelial plug with numerous beta-galactosidase-positive cells (h), accumulation of milk in the lactiferous sinus and only a few PCNA-positive cells. Note the intense beta-galactosidase staining in the keratinocytes. Original magnification X75 (a, b, d, e, g, h, j and k) X150 (c, f, I and l).

**Figure 7:**
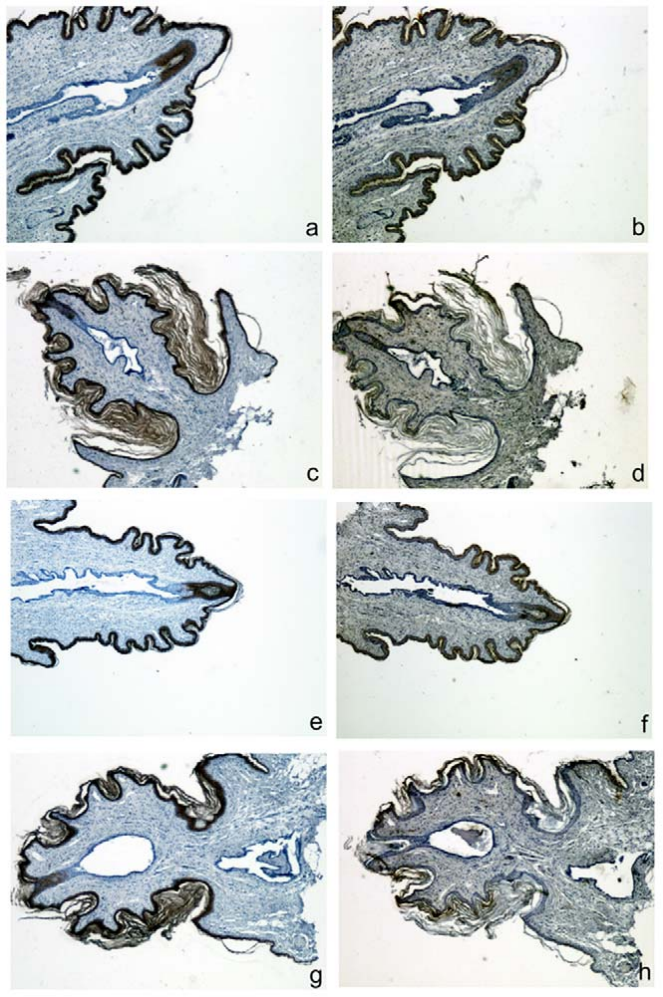
Nipples from nm23-M1-/- females present normal epidermis. Serial sections of nipples were stained with anti-K10 (a, c, e and g) and anti-K14 (b, d, f and h). Sections from WT (a, b, c and d) and mutant (e, f, g and h) nipples show no difference in K10 or K14 signal, whether they were retrieved from nursing (a, b, e and f) or not nursing (c, d, g and h) females. Original magnification X75.

Nm23-M1^−/−^ mutant glands displayed delayed but normal development and apparently normal function until L1. Despite that the females were unable to nurse their pups, which died from dehydration early after birth. Nipples were present and apparently suckled by the pups. Therefore, we hypothesized that the milk was not ejected from the gland itself. Indeed, as already mentioned, RNAs encoding milk proteins were evidenced and we could see the milk itself in the lumen of the lobuloalveolar structures (see Figure 3b). In particular in several instances, we observed distortions of the alveolae in the mutant glands (see Figure 3d). This suggests that the milk was accumulating in the ducts. In the mouse, there is only one lactiferous sinus exiting the gland through the nipple [24]. During pregnancy, the nipples undergo changes in size and in the architecture of subjacent connective tissue. The dermis gets looser with extensive remodeling of collagen bundles and elastic fibers and the epidermis wrinkles, showing thickened stratum corneum. Macroscopic comparison of mutant and WT nipples showed that the size of the nipples, in particular their width and length appeared normal in the nm23-M1^−/−^ females (data not shown). In addition, for early post-partum females, the nipples seemed to be suckled by the newborns before they died. When nipple sections were observed, the general structure of the nipples was similar in mutant and WT mice (Figure 5a and b). In particular, the connective tissue maturation of the nipples seemed unaffected. However, as mentioned before, we observed accumulation of the milk in the lactiferous duct of the mutant nipples (Figure 5b). Thus, we performed longitudinal serial sections of the nipples in order to examine the opening at the very extremity of the lactiferous sinus. The epithelium of the lactiferous sinus appeared thinner in mutants as compared to WT nipples. More interestingly, we observed a stenosis in the nm23-M1^−/−^ nipples (n = 4 for the mutant nipples and n = 3 for the WT, Figure 5c and d), obstructing the terminal end of the lactiferous duct. To further explore the structure of the lactiferous sinus at the tip of the nipple, we performed immune-staining of longitudinal serial sections of nipples with antibodies directed against the Proliferating Cell Nuclear Antigen (PCNA) to assess cell proliferation, against the beta-galactosidase, present only in the nuclei of the nm23-M1^−/−^ cells, and the keratine 2e (K2e), a specific marker of the nipple epidermis [25]. As we could not evidence any apoptotic figures, possibly because of the very transient existence of apoptic bodies, we did not quantify apoptosis by apoptosis-specific labeling. We also checked the expression of both basal and suprabasal layers markers of the epidermis, namely the keratin 14 (K14) and 10 (K10). Expression of K2e appeared similar in the nm23-M1^−/−^ nipples as compared to wild type, regardless of the feeding status of the females (Figure 6 last column). Similarly, the expression and location of the epidermis markers K10 and K14 seemed unchanged (Figure 7). Interestingly, these markers clearly showed that the cell plug derived from the lactiferous sinus extremity but not from the epidermis (Figure 7). Counting of PCNA-positive cells within the lactiferous sinus directly below the final opening revealed that nipples from WT feeding females displayed active proliferation of cells along the duct, phenomenon which was greatly attenuated in nipples from nm23-M1^−/−^ females failing to feed their pups (Figure 6, Table 1). Remarkably, the proliferating cells were arranged along the duct in the cell layer opposite to the lumen (Figure 6). Very interestingly, nipples from WT females which were not feeding at L3 (because their pups had been removed at L1) showed staining and structure very similar to that of mutant females. Indeed, cell proliferation was about 2-fold lower as compared to WT females (Figure 6, Table 1), the stenosis was present and the milk was accumulating in the duct (Figure 6). Conversely, the nipples from nm23-M1^−/−^ females which were able to feed their babies (about 15% of the nm23-M1^−/−^ females, as already mentioned) showed structure and staining very similar to that of WT females (no stenosis, and about 30% of PCNA-positive cells, Figure 6, Table 1). Finally, we analyzed nuclear beta-galactosidase signal, which is only present in the nm23-M1-expressing cells. The majority of the cells within the epithelial plug that blocked the lactiferous duct were positive for beta-galactosidase signal, suggesting that nm23-M1-positive cells persisted in the defective nipples (Figure 6).

**Table 1:** PCNA indexes in lactiferous sinuses.

Genotype	% PCNA positive cells	P values
Wild type feeding	35.26±0.09 (1597)	NA
Wild type not feeding	15.57±0.05 (781)	0.006
Nm23-M1-/- feeding	32.18±0.05 (1715)	0.9
Nm23-M1-/- not feeding	19.05±0.07 (1345)	0.03

Longitudinal sections of L3 nipples were stained for PCNA detection and the number of PCNA-positive nuclei over the total number of nuclei in the lactiferous sinus was counted in at least 4 fields for 3 to 5 independent nipples in each group (except for the Nm23-M1-/- feeding group, n = 2). Results are expressed as means ±SD. The number of total counted nuclei is indicated in parenthesis. P values were obtained by unpaired *student t tests* against the wild type feeding values. NA: not applicable.

Thus, the defect of nursing ability of the nm23-M1^−/−^ females seems to be caused by a deficient terminal maturation of the lactiferous duct, resulting in milk accumulation in the duct and failure in final milk ejection.

**Figure 8:**
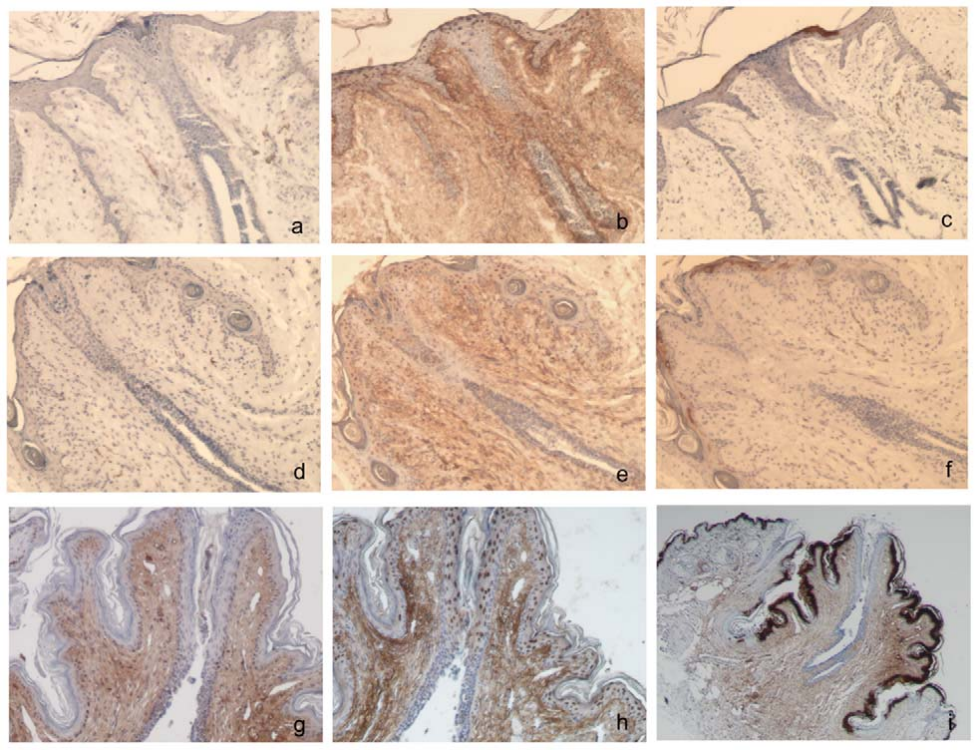
Nipples from nm23-M1+/− virgin females present an epithelial plug rich in beta-galactosidase positive cells. Serial sections of nipples from 6 weeks old (a, b and c), 12 weeks old (d, e and f) and L1 lactating (g, h and i) nm23-M1+/− females were stained with anti-PCNA (a, d, and g), anti-beta-galactosidase (b, e and h) and anti-K2e (c, f and i). Sections from 6 and 12 weeks old display an epithelial plug at the edge of the lactiferous sinus with numerous apoptotic figures (a) and beta-galactosidase-positive cells (b,e). Very few PCNA-positive cells could be seen (a, d). In the nipple from L1 lactating heterozygous females, PCNA-positive cells appeared more numerous and beta-galactosidase-positive cells disappeared together with the epithelial plug. Original magnification X150 except for i: X75.

To gain insight into the maturation of the lactiferous sinus and the relationship with nm23-M1, we performed PCNA and beta-galactosidase staining of longitudinal serial sections from 6 week-old and 12-week-old nm23-M1^+/−^ heterozygous virgin females. Nipples from younger females were too underdeveloped to gather reliable information. Strikingly, nipples from virgin females displayed very few PCNA-positive cells and the stenosis was easily evidenced at both ages (Figure 8). In the region of the epithelial plug, numerous beta-galactosidase-positive cells could be detected, along with apoptotic figures, especially at 6 weeks old (Figure 8). The same experiments were carried out on nipples from m23-M1^+/−^ heterozygous feeding females at L1. As expected, no stenosis was observed and PCNA-positive cells were detected along the lactiferous ducts, although less numerous than at L3. In addition, very few beta-galactosidase-positive nuclei were present. Thus, these results are consistent with the observation made at L3 in nipples from non feeding females.

## Discussion

The generation of nm23-M1 deficient mice was first designed to explore the role of the gene as a suppressor of metastasis. We did not expect that a lethal phenotype would affect the mutant newborns. The present study sought to better understand this mortality. Our main findings are: (1) lack of nm23-M1 leads to reversible mammary gland growth retardation in virgin mutant females and (2) although displaying a normal mammary gland function, nm23-M1^−/−^ females are not able to feed their babies because the terminal end of the lactiferous duct is obstructed in the nipple. We provide evidence showing that the growth retardation of the virgin gland is connected to abnormal proliferation and apoptosis in the terminal end buds, and we discuss some of our hypothesis to explain the deficient final maturation of the lactiferous duct in the mutant nipples.

It is noteworthy to mention here that despite the well-accepted implication of nm23-1 gene in the control of breast cancer metastasis, mutant females kept in our animal facility for up to 18 months (virgin or not) did not display a higher rate of mammary gland tumors than their WT counterparts.

As expected, nm23-M1 gene expression was evidenced in the epithelial compartment of the mammary gland. Nm23 genes were early observed in epithelial cells developed from mammary gland tumors [2]. This pattern has been confirmed in many studies ever since. So if any defects were related to lack of nm23-M1 in the mammary gland, they would more likely manifest in the mammary ducts morphology or function.

The growth retardation observed in nm23-M1^−/−^ virgin females was associated to reduced proliferation and apoptosis in the TEBs. The nm23-1 gene displays a confusing multifaceted role in the control of cell proliferation and apoptosis. For example, a recent study showed that nm23-H1, encoding the human NDPKA isoform, positively regulated the proliferation of the K562 cell line by keeping the cells cycling [26]). Conversely in another B cell line, nm23-H1 had the reverse effect [27]. Most of the studies have been initially carried out in vitro on transformed cell lines. Typically, forced expression of the nm23-1 gene did not affect tumorigenicity and cell proliferation but instead cell motility and tumor invasiveness capacities [28], especially in the pathological mammary glands. Moreover, nm23 genes studies were first focused on the correlation between nm23 genes expression and cancer initiation and progression [29]. We report for the first time an effect of the lack of nm23-1 gene in physiological conditions. Our work shows that in mammary glands, when present, nm23-1 gene controls positively the proliferation. This could be directly linked, as in cancer, to nucleotides metabolism. However, this effect seems minor since the tissue overcomes the growth retardation and produces a functional mammary gland in mutant females. Thus, since our main goal was to establish the reason why the mothers were unable to nurse, we did not pursue molecular exploration of proliferation in the mutant virgin glands. Furthermore, it would be of great interest to assess cell proliferation in other tissues and in normal conditions in the mutant mice. In that matter, we have explored the distribution of cells in hematopoietic compartments and found no difference in nm23-M1^−/−^ mice as compared to wild type mice (S. Dabernat and Z. Ivanovic, personal communication). Thus, it seems clear that the diverse effects carried by the nm23-1 gene are highly dependent on the cell type and the physiological status of the model. The same assessment can be drawn when apoptosis is explored in relation with nm23 genes activity. Interestingly, nm23-H1 is a granzyme A-induced DNase involved in the positive control of apoptotic response to cytotoxic T lymphocyte activation [30]. In addition, nm23-H1 is a negative regulator of the Macrophage Induced Factor (MIF), a cytokine with anti-apoptotic properties, in particular in breast cancer cells [31], [32]. Besides, nm23-H1 positively regulated the p53-induced apoptosis pathway with STRAP (serine-threonine kinase receptor-associated protein) [33]. Collectively with the data presented here, these observations suggest a pro-apoptotic effect of the nm23-1 gene in the developing mammary gland. We found that the balance of members of the BCL2-like family was in favor of cell survival in the mutant glands. This was further confirmed with an elevation of the expression of AKT and of NFkB (not shown). It seems likely that the mechanism of apoptosis regulation by nm23-M1 might impact several pathways.

The most striking phenotypical trait of the nm23-M1^−/−^ mice was the high mortality rate of newborn pups from homozygous mutant females. The mutant mothers exhibited normal nursing behavior, such as nesting and pup retrieval but they did not feed the pups and no pups survived for more than 24–36 hours in the majority of the mutant litters (85% of the litters). As we observed growth retardation of the mammary gland of virgin females, we expected to find underdeveloped glands during gestation and at the onset of lactation. However, no gross morphological abnormality was evidenced in whole mount mammary gland preparation. Lactation defects can be very picky and tedious to analyze. We explored most of the aspects of lactation as recommended by experts [34]. Our attempt to identify deficient functions of the mutant glands failed and we clearly evidenced milk, RNAs encoding milk protein and milk proteins themselves. The signaling pathway controlled by the main milking hormone, the prolactin, was active, oxytocin did not rescue the mutant mother feeding capacities and oxytocin receptor and myoepithelial cells, responsible of milk ejection were easily evidenced. It was difficult to characterize the function of the mutant mammary gland as compared to the WT because the pups from the nm23-M1^−/−^ females died unfed within the first 2 days after birth and the mammary glands started the process of involution. However, we made clear that the involution process was not to be accounted for the nursing defect. Indeed, WT glands, prevented from feeding, underwent similar morphological and molecular changes as those observed in the mutant glands.

Since we evidenced milk in the lumen of the lobuloalveolae, we hypothesized that the final ejection of the milk, through the nipple and outside of the body did not occur. Very few studies report a phenotype similar to that of nm23-M1^−/−^ mice, where dysfunction of the nipples is evidenced, and is the cause of feeding defects. In the rodents, nipples undergo histological changes during the reproductive cycle [24]. In relaxin or relaxin receptor (LGR7) mutants, nipples are underdeveloped and the characteristic histological shift is impaired [35], [36]. Similar observations were made in mice lacking the PTH/PTRH (parathyroid hormone/parathyroid hormone-related protein receptor) pathway [37]. The nm23-M1^−/−^ mutant nipples appeared histologically normal when observed in transversal sections (not shown), in particular, the histological hallmark of nipple maturation was present and appeared normal. The only option left was to perform serial longitudinal sections of the very final end of the lactiferous duct at the very extremity of the nipple. There is only one lactiferous duct at the extremity of the rodents nipples [38]. We found that an epithelial cell plug was blocking the final opening of the duct preventing the milk from flowing out. We also noticed that the thickening of the lactiferous epithelium, which is a phenomenon described in rodents [38] was decreased in the mutant nipple as compared to the feeding WT. Besides, the epidermis layers seemed normal in the mutant nipples. When we analyzed nipples from WT females that were not nursing, we found that they shared the same morphology as the mutant ones. Similarly, the few mutant nipples analyzed from feeding females appeared undistinguishable from the WT ones. To our knowledge, very little information is available on the maturation of the lactiferous ducts. In the mouse nipples, proliferation takes place during pregnancy [24]. We made similar observations since lactiferous sinuses from virgin females displayed very few PCNA-positive cells and about 30% of the cells from lactiferous ducts appeared actively proliferating in nipples from feeding females. We evidenced that this phenomenon was part of the nipple maturation process since it was not observed in nipples from non feeding WT females. Moreover, we observed that lactiferous sinuses from virgin females contained an epithelial plug and apoptotic bodies were present around the lumen. We believe that as for the lumen of lobuloalveolar units, it is very likely that the lumen of the terminal lactiferous duct appears by cavitation i.e. the apoptosis induction of the inner cell population [20], [39]. In the mutant mice, this phenomenon might be inhibited or delayed during a period long enough to cause the pups death by starvation. This hypothesis is further strengthened by the observation of delayed apoptosis in the developing virgin gland and by the presence of beta-galactosidase-positive cells within the persisting epithelial plug in the nipples from females that failed in nursing their pups.

In conclusion, our work evidenced that nm23-1 gene affects the proliferation/apoptosis balance of the developing mammary gland, which delays the maturation of the virgin gland. The further maturation of the glands during pregnancy did not seem overly affected, neither the function of the lactating glands. However, as milk is produced, its delivery out of the nipple was prevented by the persistence of an epithelial plug.

## Supporting Information

Figure S1 **nm23-M1 gene is expressed within the luminal cells of the mammary glands epithelium.** Sections of mammary glands from 6 weeks old virgin (a) and of L1 lactating nm23^+/-^ females (b) have been processed to detect in situ expression of LACZ. In both sections, beta-galactosidase is detected in the epithelium of the glands, the surrounding myoepthelial cells and adipocytes being negative (original magnification X400).
(TIF)

Figure S2 **Newborn nursed by nm23-M1^−/−^ females display empty stomach and die shortly after birth.** Independent of the newborn genotype or gender, babies nm23-M1^+/−^ nursed by nm23-M1^−/−^ females do not display milk in their stomach (b, white arrow), which can be easily seen in babies nm23-M1^+/−^ fed by WT females (a, dashed white circle).
(TIF)

Figure S3 **Oxytocin receptor is normally detected in the nm23-M1^−/−^ mammary gland.** Sections from intestine (a) stained with anti-OTR antibody were used as a negative control tissue and uterus (b) as a positive control. Analysis of mammary gland (MG) sections from WT (c) and nm23-M1^−/−^ (d, KO) stained with anti-OTR antibody revealed normal pattern of OTR in the mutant glands. Original magification X400.
(TIF)
