# Supplementary figures and images for: The Anti-Metastatic nm23-1 Gene Is Needed for the Final Step of Mammary Duct Maturation of the Mouse Nipple
Source: PLoS One. 2011 Apr 7;6(4):e18645. doi: 10.1371/journal.pone.0018645 (PMC3072419; doi:10.1371/journal.pone.0018645)

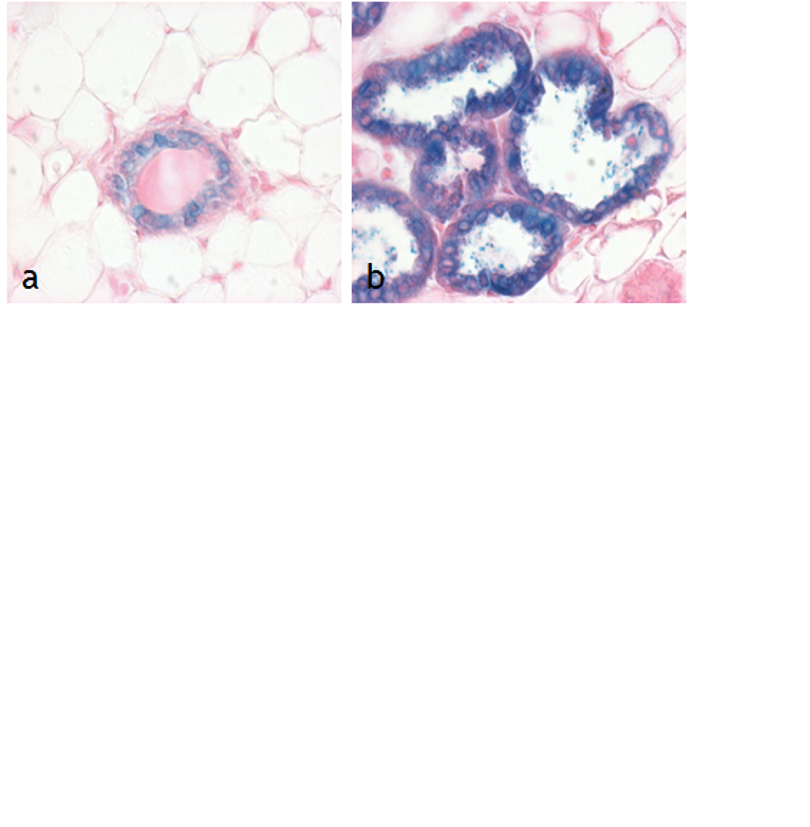

Supplement: Figure S1 — nm23-M1 gene is expressed within the luminal cells of the mammary glands epithelium. Sections of mammary glands from 6 weeks old virgin (a) and of L1 lactating nm23+/- females (b) have been processed to detect in situ expression of LACZ. In both sections, beta-galactosidase is detected in the epithelium of the glands, the surrounding myoepthelial cells and adipocytes being negative (original magnification X400). (TIF) [file pone.0018645.s001.tif]

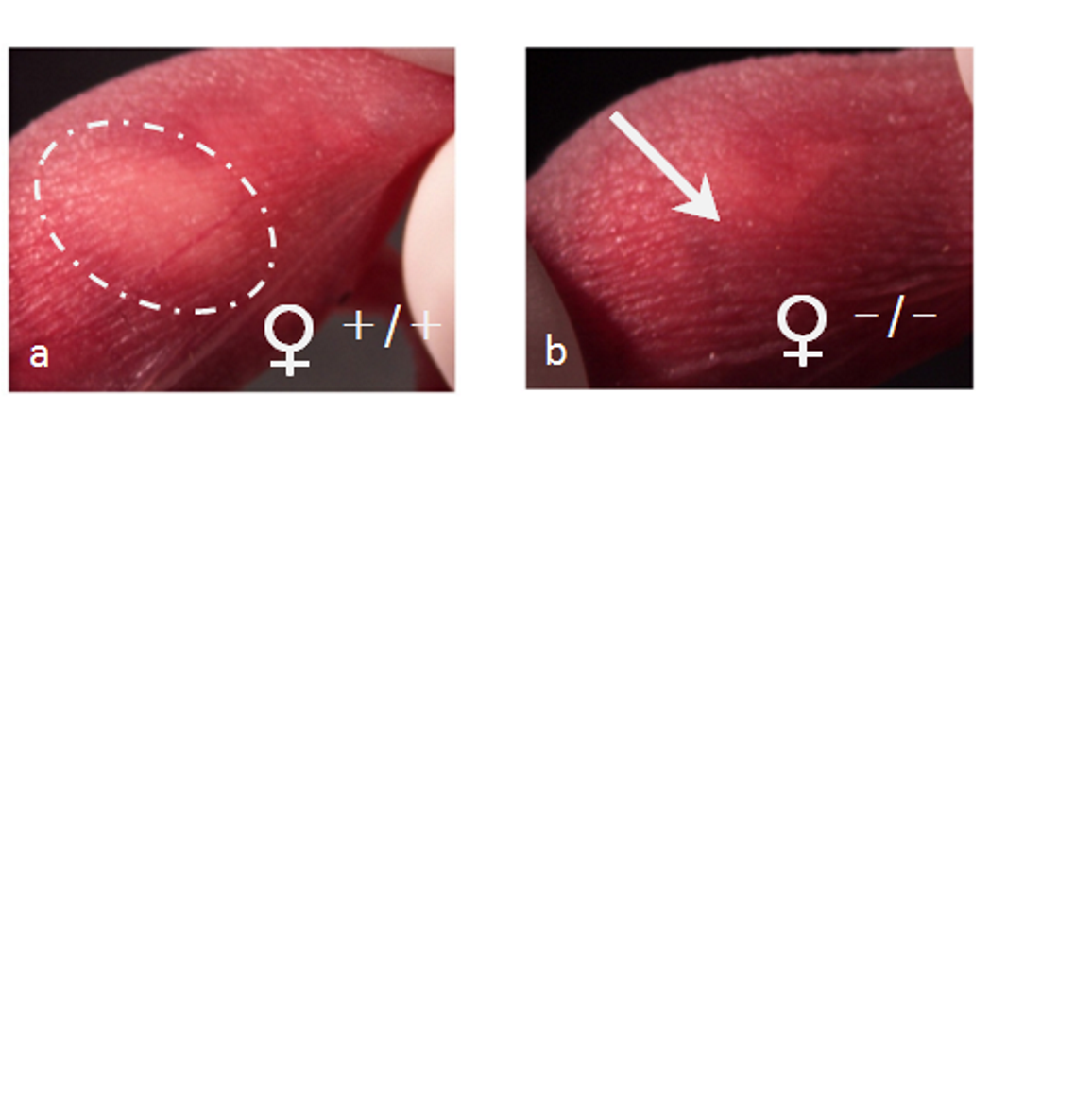

Supplement: Figure S2 — Newborn nursed by nm23-M1−/− females display empty stomach and die shortly after birth. Independent of the newborn genotype or gender, babies nm23-M1+/− nursed by nm23-M1−/− females do not display milk in their stomach (b, white arrow), which can be easily seen in babies nm23-M1+/− fed by WT females (a, dashed white circle). (TIF) [file pone.0018645.s002.tif]

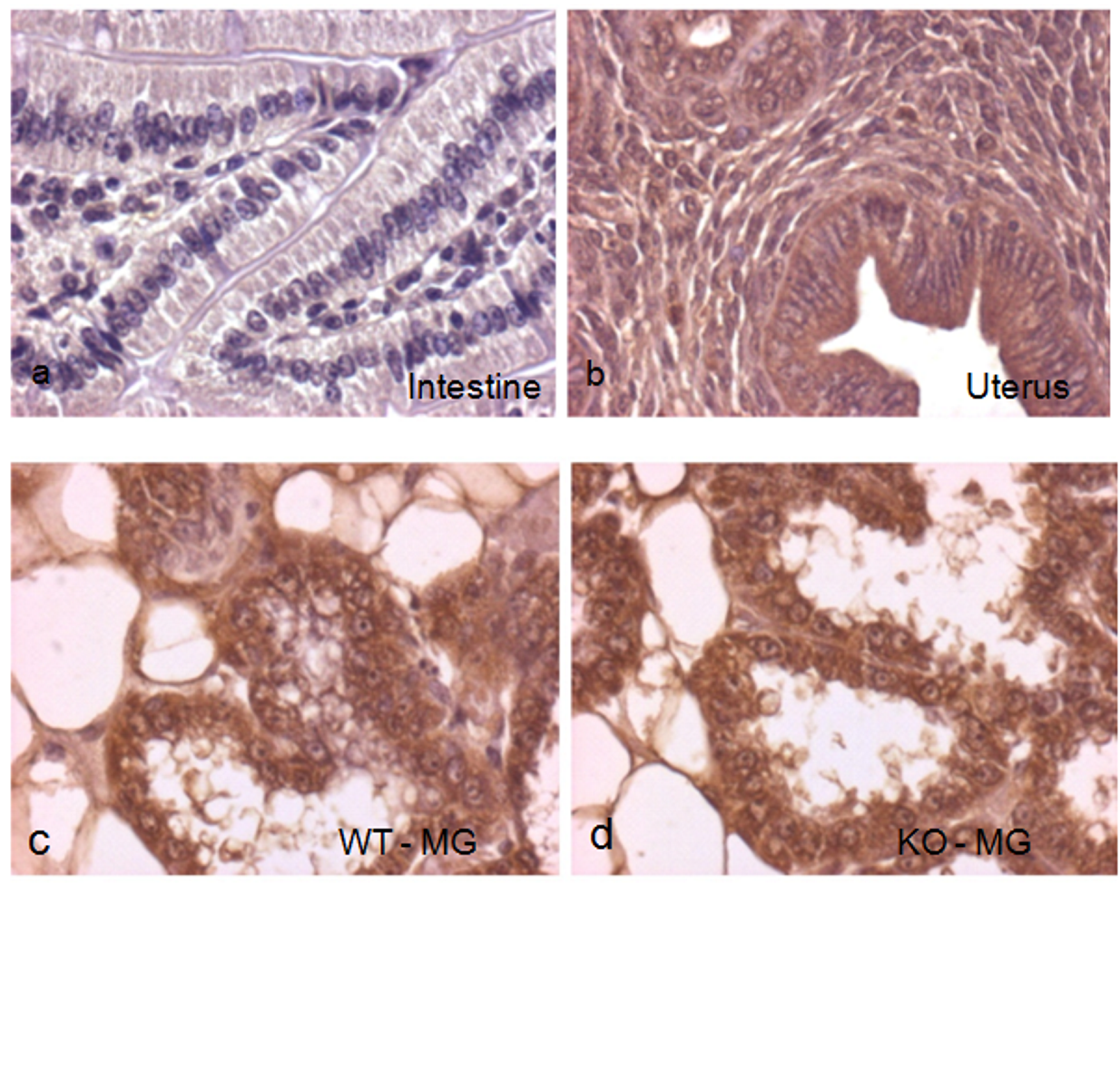

Supplement: Figure S3 — Oxytocin receptor is normally detected in the nm23-M1−/− mammary gland. Sections from intestine (a) stained with anti-OTR antibody were used as a negative control tissue and uterus (b) as a positive control. Analysis of mammary gland (MG) sections from WT (c) and nm23-M1−/− (d, KO) stained with anti-OTR antibody revealed normal pattern of OTR in the mutant glands. Original magification X400. (TIF) [file pone.0018645.s003.tif]
